# The coronoid as the key fragment of trans-ulnar fracture–dislocations of the elbow: Insights from a retrospective cohort comparison using the coronoid-centric Mayo classification system

**DOI:** 10.1186/s10195-026-00921-x

**Published:** 2026-04-21

**Authors:** Leopold Henssler, Maximilian Kerschbaum, Miriam Zanklmaier, Leon Alexander Wieczorek, Volker Alt, Lisa Klute

**Affiliations:** Department of Trauma Surgery, University Hospital Regensburg, Franz-Josef-Strauß-Allee 11, 93053 Regensburg, Germany

**Keywords:** Elbow, Trauma, Olecranon, ORIF, Trauma, Fracture, Monteggia, Trans-olecranon

## Abstract

**Background:**

Trans-ulnar fracture–dislocations of the elbow are rare injuries with complex fracture patterns and variable outcomes. Traditional classification systems offer limited prognostic value. A recently introduced coronoid-centric Mayo classification distinguishes injury subtypes based on coronoid attachment and identifies trans-ulnar basal coronoid (TUBC) fractures as a particularly challenging entity. This study evaluated outcomes across Mayo fracture types and explored factors associated with inferior results in TUBC injuries.

**Materials and methods:**

In this retrospective cohort study, surgically treated trans-ulnar elbow fracture–dislocations managed at a level I trauma center between 2010 and 2022 were identified and classified according to the Mayo system. Demographic data, injury characteristics, surgical management, radiographic outcomes, and complications were recorded. Functional outcomes were assessed after a minimum follow-up of 12 months using the Mayo Elbow Performance Score (MEPS); Oxford Elbow Score (OES); Quick Disabilities of Arm, Shoulder and Hand Questionnaire (QuickDASH); European Quality of Life Five-Dimension, Five-Level Version (EQ-5D-5L); and range-of-motion measurements. Radiographs were analyzed for union, instability, heterotopic ossification, and post-traumatic osteoarthritis (OA).

**Results:**

A total of 52 patients were included (14 trans-olecranon, 28 TUBC, 10 Monteggia-variant). TUBC injuries were the most common subtype (53.8%). Post-traumatic OA was significantly more frequent in TUBC injuries than in fractures with coronoid attachment to a major fragment (88% versus 61%, *P* = 0.047). Higher-grade OA and persistent instability were associated with inferior functional outcomes. Although functional scores tended to be lower in TUBC injuries, differences compared with other subtypes were not consistently statistically significant. Within the TUBC group, poorer outcomes were observed when stable screw fixation of the basal coronoid fragment could not be achieved.

**Conclusions:**

TUBC fracture–dislocations represent a high-risk subgroup of trans-ulnar elbow injuries. Stable fixation of the coronoid base appears critical for achieving favorable outcomes. The Mayo classification provides clinically relevant stratification and prognostic insight for these complex injuries.

*Level of evidence*: Level IV.

## Introduction

Trans-ulnar fracture–dislocations of the elbow are relatively rare and complex injuries and show highly variable outcomes due to diverse fracture patterns [1–5]. Historically, fracture–dislocations involving the dorsal cortex of the ulna have been classified either as “trans-olecranon” or “Monteggia-variant” fracture–dislocations depending on the integrity of the proximal radioulnar joint [6, 7], since Monteggia claimed a Monteggia fracture to be a fracture of the proximal ulna with disrupture of the proximal radioulnar joint and radial head dislocation [7]. Bado divided the “Monteggia-variant” fractures into four subtypes according to the direction of the dislocation [8], and Jupiter later subdivided the most frequent Bado Type II injuries with posterior dislocation of the radial head according to the fracture pattern of the proximal ulna [7, 9], resulting in the most frequently used classification system over the following decades. However, this classification system has limited applicability in fractures with an intact proximal radioulnar joint and yet dislocation of the radial head or fractures involving a comminuted fracture area around the basal coronoid and the lesser sigmoid notch of the proximal ulna since the integrity of the proximal radioulnar joint cannot always be determined in these cases. Moreover, this classification system only describes fracture patterns, but neither treatment strategies nor prognostic value can be extracted from the fracture types.

Therefore, different classification systems have tried to further describe injury patterns and have identified key fragments to be addressed in the surgical treatment of elbow fracture–dislocations [10–14]. The Montecranon classification by Spross et al. [14] divides fractures in four subtypes depending on whether coronoid fractures, ligaments, or radial head fractures need to be addressed besides the proximal ulnar fracture and do not distinguish whether the ulnar fracture involves the olecranon or meta-diaphyseal zone. Using the same key components of the injury, Bagga et al. [11] proposed the Coronoid, Proxima Ulna, Radius, and Ligaments (CURL) classification, in which the coronoid, ulna, radius, and ligaments are each assigned a numerical value between 0 and 2, depending on whether the components are uninjured or have simple or complex injuries. The Wrightington classification not only refers to trans-ulnar but includes all fracture–dislocations of the elbow and builds on the three-column concept of elbow stability [10, 15, 16]. It divides the injuries into six subtypes, where only types B and D represent trans-ulnar fracture–dislocations.

However, while most of these classification systems provide reasonable help in preoperative planning of surgical treatment of these injuries [11, 14, 17], they are too detailed for consistent use in clinical practice or research. Therefore, the department of orthopedic surgery of the Mayo Clinic defined a new classification [18] that focuses on the coronoid and divides trans-ulnar fracture–dislocations into three major subgroups according to the attachment of the coronoid to either the ulnar shaft (“trans-olecranon fracture–dislocations”) or the olecranon (“Monteggia-variant fracture–dislocations”). Fracture patterns in which the coronoid process is not attached to either the ulnar shaft or the olecranon were termed “trans-ulnar basal coronoid” (TUBC) fractures [18]. These fractures correspond to the previously described “basal coronoid fractures, subtype 2” according to O’Driscoll’s coronoid classification [19] and types IIA or IID according to Jupiter’s classification of posterior Monteggia-like fracture–dislocations [9]. The authors observed that the newly defined trans-ulnar basal coronoid type is the most difficult to treat, with a high rate of complications and revision surgeries [5, 18, 20]. With excellent inter- and intra-rater agreement [18], the Mayo classification offers everything required for broad application in science and everyday clinical practice in the future: it facilitates uniform communication, directly determines the treatment strategy, and enables prognostic assessment.

Therefore, the objective of the present study was to evaluate outcome of the different fracture types according to the new classification system and identify possible reasons for limited outcome of TUBC fractures.

## Methods

### Patient selection

This prospective pilot study was conducted at a level I trauma center in accordance with the Declaration of Helsinki, following approval by the Local Ethics Committee of the University of Regensburg (Number: 23-3228-104). All patients admitted between January 2010 and December 2022 with one of the following International Classification of Diseases Version 10 (ICD-10) admission codes were identified: S52.01, S52.02, S52.09, S52.1, S52.21, S53.0, S53.1, S53.2, and S53.3. A total of 635 patients were identified, of whom 582 met the inclusion and exclusion criteria. Exclusion criteria were elbows that had the index surgery elsewhere, injuries sustained prior to the study period, pathological fractures, incomplete/inappropriate imaging, and age < 16 years at the time of injury.

**Fig. 1:**
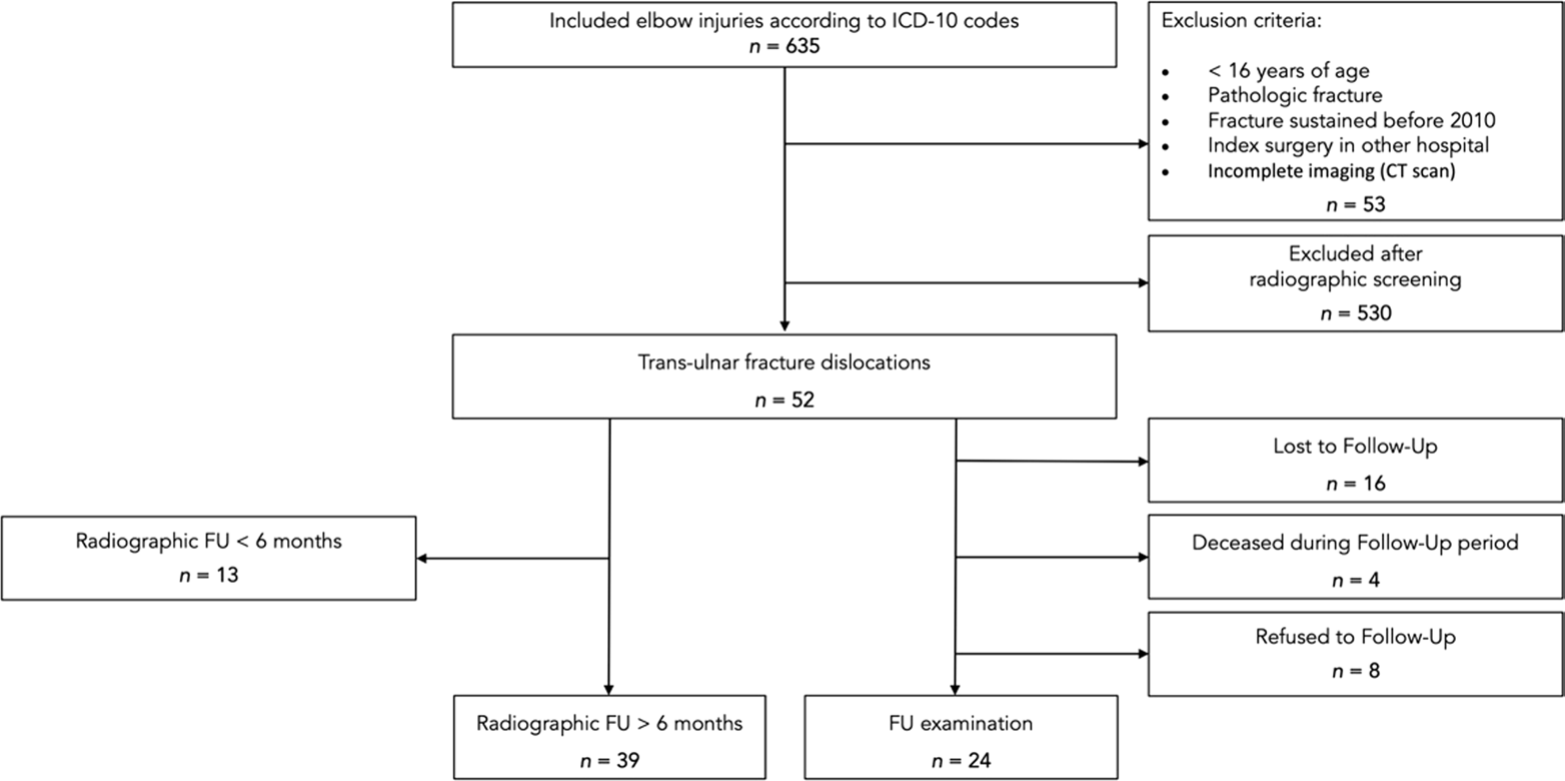
Strengthening the Reporting of Observational Studies in Epidemiology (STROBE) flow diagram of patients included in the analysis

The eligible cohort was retrospectively reviewed by two experienced orthopedic trauma surgeons using available radiographic imaging and medical records. All trans-ulnar elbow fracture–dislocations were extracted from the cohort and classified according to the recently published coronoid-centric Mayo classification system [5, 18]. A total of 52 patients were identified with trans-ulnar fracture–dislocations of the elbow (Fig. 1). Of these patients, baseline demographic data and radiographic outcome were recorded. Subsequently, patients were prospectively contacted to gather patient reported outcomes after minimum follow-up (FU) of 12 months.

### Surgical management

All 52 patients were operatively treated via a posterior approach in supine position with the arm positioned on an arm table. The majority of ulnar fractures (46 patients, 88.5%) were fixed using a single posterior anatomically preshaped olecranon plate (3.5 mm LCP^™^ Olecranon Plate, DePuy Synthes, Raynham, Massachusetts, USA), while tension band wiring was used in 4 patients and double plating in 4 patients. In TUBC fracture–dislocations, the basal coronoid fragment was fixed with separate lag screws in 12 elbows, lag screws through the plate in 10 elbows, and transosseous suture fixation in 3 elbows. In another three elbows the basal coronoid fragment was not adequately addressed.

Radial head or neck fractures were observed in 37 patients. If stable reconstruction was deemed feasible, internal fixation by screws or plate was performed (*n* = 20). If no stable reconstruction was possible, the threshold was set low for primary radial head arthroplasty for more primary stability (*n* = 9). In eight patients, smaller fragments without relevance for radial column stability were resected without additional fixation. For stability reasons, none of the elbows received total resection of the radial head as a primary fracture treatment. The lateral ulnar collateral ligament received repair in 17 elbows. For treatment of the radial head, an additional radial approach using the Kocher or Kaplan interval was used in 27 of 37 patients with a concomitant radial head or neck fracture while the remaining 10 patients were treated via the dorsal approach (modified Boyd’s approach) only.

Due to persistent instability following surgical fixation, a hinged external elbow fixator was necessary in five elbows.

### Radiographic assessment

**Fig. 2:**
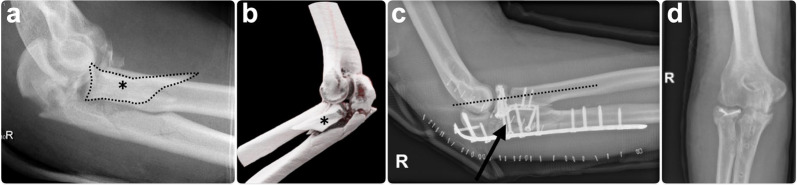
Example of radiographic follow-up of a trans-ulnar basal coronoid (TUBC) fracture–dislocation. **a** A 36-year-old male patient sustained a trans-ulnar basal coronoid fracture–dislocation of the elbow with a large basal coronoid fragment (dashed line) following a high-energy motor vehicle accident. **b** Three-dimensional CT reconstruction demonstrates displacement of the basal coronoid fragment (^*^) with associated ulnohumeral subluxation. **c** Postoperative radiographs after dorsal plate fixation of the ulna and additional deep lateral approach for radial head plate fixation show a centered radiocapitellar alignment without signs of persistent instability. In this case, the basal coronoid fragment was fixed through the plate with additional lag screws (arrow). **d** Radiographs obtained 18 months postoperatively, after implant removal, demonstrated post-traumatic osteoarthritis of the ulnohumeral joint

Of all 52 elbows, those with at least 6 months radiologic FU underwent standardized radiographic evaluation. Radiographs were assessed for signs of instability (joint incongruency or a decentered radiocapitellar axis), rates of union, Hastings and Graham heterotopic ossification (HO) grade [21], and Broberg and Morrey arthritis grade [22] (Fig. 2).

### Clinical assessment

Patients first underwent a standardized clinical examination. Elbow range of motion was documented using a goniometer in extension/flexion and pronation/supination according to the neutral-zero method, and clinical stability testing of the elbow was performed. To allow for comprehensive evaluation of functional outcomes, two commonly reported elbow-specific scoring systems were applied: the Oxford Elbow Score (OES) and the Mayo Elbow Performance Score (MEPS). In addition, the Quick Disabilities of Arm, Shoulder, and Hand score (QuickDASH) was used to assess global upper extremity function, and the European Quality of Life Five-Dimension, Five-Level Version (EQ-5D-5L) was administered to evaluate health-related quality of life.

### Statistical analysis and reporting of results

Statistical analyses were conducted using IBM SPSS Statistics (version 29; IBM Inc., Armonk, NY, USA), with significance set at *P* < 0.05. Pairwise deletion was used to handle missing data. Continuous data were analyzed and presented as the median and the range. Categorical data were presented as the total count and percentage. Normality of the data was assessed using Shapiro–Wilk testing. Following, for comparison of two groups the Mann–Whitney *U* test was used, and comparisons of more than two groups were performed by the Kruskal–Wallis test. Chi-squared test was used for comparison of the distribution of patient characteristics. All results were reported according to the Strengthening the Reporting of Observational Studies in Epidemiology (STROBE) guidelines.

## Results

### Patient epidemiology

Of the eligible 52 patients, 10 fractures (19.2%) were classified as Monteggia-variant (MV) fracture–dislocations, 14 fractures (26.9%) were classified as trans-olecranon (TO) fracture–dislocations, and 28 fractures (53.8%) were classified as trans-ulnar basal coronoid (TUBC) fracture–dislocations. TO fracture–dislocations were observed in a significantly younger patient population (*p* = 0.022) and presented significantly less often with proximal radial fractures (*p* < 0.001) than with TUBC or MV fracture–dislocations. Differences in sex and high-energy trauma as a causal mechanism were not significantly different (Table 1).

**Table 1:** **Patient characteristics**. While TO injuries occurred in younger patients and were least frequently associated with radial head fractures, MVs were observed in an older population and were significantly more frequently associated with radial head fractures. Significant differences are indicated by bold p-values. TO = trans-olecranon, TUBC = trans-ulnar basal coronoid, MV = Monteggia-variant

	TO (*n* = 14)	TUBC (*n* = 28)	MV (*n* = 10)	*p*-Value
Age, years (range)	38 (16–70)	53 (17–76)	61 (43–71)	0.022^*^
Sex (female)	5/14 (36%)	12/28 (43%)	8/10 (80%)	0.073^**^
High-energy trauma	11/14 (79%)	15/28 (54%)	3/10 (30%)	0.058^**^
Proximal radial fracture	4/14 (29%)	24/28 (86%)	9/10 (90%)	< 0.001^**^

Statistically significant differences are marked by bolt *p*-values

^*^Kruskal–Wallis test

^**^Chi-squared test

### Radiographic outcome

A minimum of 6 months of radiographic data were available for 39 elbows (9 TO, 24 TUBC, and 6 MV fracture–dislocations; FU rate 75%). Neither age, sex distribution, nor fracture pattern differed significantly between patients who were available for radiographic FU assessment and those who were not (*p* > 0.05 each).

Radiographs were evaluated after a median of 30.9 (range 6.1–146.6) months, and radiographic FU did not differ between the groups (*p* = 0.886). Only three injuries (all TUBC) did not show complete radiographic healing after 7.5 months, 10.8 months, and 78.5 months, respectively.

Heterotopic ossifications (HO) were present in 33% (3/9) of TO cases, 42% (10/24) of TUBC cases, and 67% (4/6) of MV injuries (*p* = 0.423). However, HO causing functional restrictions (Hastings/Graham grade ≥ II) were only observed in 17% (4/24) of TUBC and 11% (1/8) of TO injuries but not in MV injuries (*p* = 0.363).

**Fig. 3:**
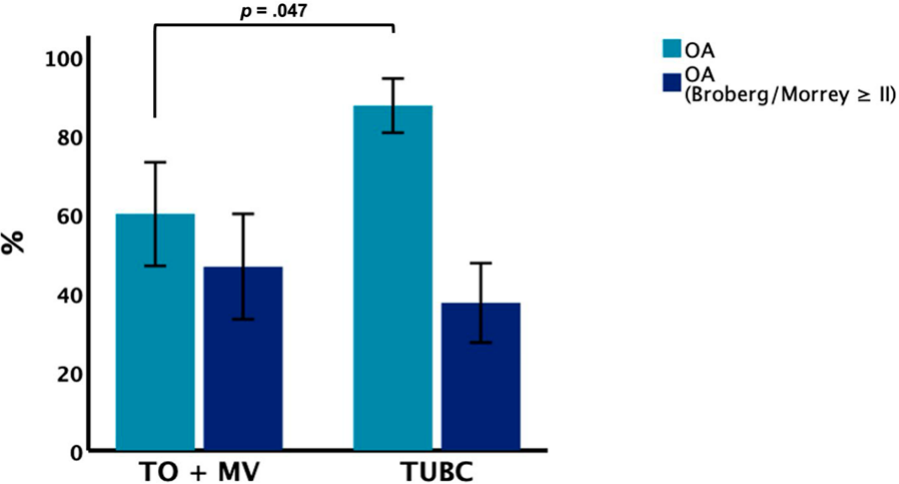
Proportion of patients presenting with post-traumatic osteoarthritis. There was a significantly higher proportion in patients with trans-ulnar basal coronoid fracture–dislocations compared with injuries without a seperate basal coronoid fragment. Error bars represent 95% CIs. *CI* confidence interval, *OA* osteoarthritis, *MV* Monteggia-variant, *TO* trans-olecranon, *TUBC* trans-ulnar basal coronoid

Elbows developed post-traumatic osteoarthritis (OA) in 56% (5/9 elbows) in TO, in 88% (21/24 elbows) in TUBC, and in 67% (4/6 elbows) in MV injuries so that post-traumatic OA was significantly more frequent (*p* = 0.047) in TUBC cases than in elbows with the coronoid still connected to one of the main fragments (MV + TO; Fig. 3). However, the prevalence of higher-grade OA (Broberg/Morrey grade ≥ II) was equally distributed between the groups (*p* = 0.571). Instability as defined by a decentered radiocapitellar line could be observed in 20.5% of cases (one TO, five TUBC, two MV), with no significant differences between the groups (*p* = 0.579).

### Functional outcome

After reaching out for eligible patients, 16 patients were lost to follow-up, 4 had died in the follow-up period, and 8 refused study participation (Fig. 1), leaving a total of 24 patients (46.2%) for functional assessment (6 TO, 15 TUBC, 3 MV). Patient and fracture characteristics did not significantly differ between patients who were followed up with and those lost to follow-up (*p* > 0.05).

**Fig. 4:**
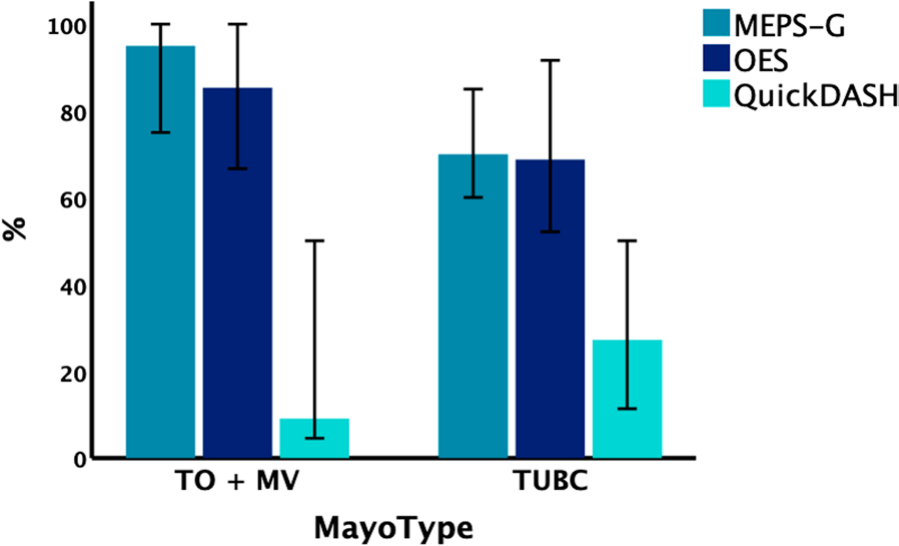
Functional outcome scores of patients with (TUBC) and without (TO + MV) coronoid base fracture. There was a trend toward poorer outcomes in TUBC injuries, but differences were not significantly significant. Error bars represent 95% CI. *CI* confidence interval, *OA* osteoarthritis, *MV* Monteggia-variant, *TO* trans-olecranon, *TUBC* trans-ulnar basal coronoid

The median time between trauma and functional re-assessment was 65.5 months (range 17–146 months) and did not significantly differ between the groups (*p* = 0.370). Of all assessed functional parameters (Table 2) only the QuickDASH showed significant differences between the groups with highest impairments in MV cases and minimal disability in TO injuries. When comparing TUBC fracture–dislocations to injuries without separate basal coronoid fragment (MV + TO; Fig. 4), functional outcome parameters were worse for TUBC injuries as measured by MEPS (median 70 versus 95 points) and OES (median 69 versus 85 points), but the differences failed to reach statistical significance (*p* = 0.086 and *p* = 0.078, respectively). In addition, compared with TO and MV patients, patients with TUBC were restricted by approximately 10 degrees in both pronation/supination and flexion arc, although the differences were not statistically significant (*p* = 0.669 and* p* = 0.893, respectively) (Table 2).

**Table 2:** Functional outcome parameters according to injury type

	Mayo type			*p*-Value
	TO (*n* = 6)	TUBC (*n* = 15)	MV (*n* = 3)	
MEPS	95 (60–100)	70 (20–100)	75 (75–80)	0.109
OES	92 (73–100)	69 (29–100)	66 (63–85)	0.072
QuickDASH	6 (0–32)	27 (0–75)	50 (43–64)	**0.022**
EQ-5D-5L	0.99 (0.70–1.00)	0.91 (0.52–1.00)	0.89 (0.18–0.91)	0.316
Flexion arc	110° (80–122°)	102° (42–138°)	112° (100–118°)	0.893
Pronation/supination arc	135° (90–170°)	125° (50–180°)	136° (80–140°)	0.669

The parameters are represented by median and ranges. Statistically significant inter-group differences (Kruskal–Wallis test) are indicated by bold *p*-values. *MEPS* Mayo Elbow Performance Score, *OES* Oxford Elbow Score, *QuickDASH* Quick Disabilities of Arm, Shoulder and Hand Score, *EQ-5D-5L* European Quality of Life Five-Dimension, Five-Level Version

Patients with higher-grade post-traumatic OA (Broberg/Morrey grade ≥ II) had significantly poorer functional outcome in follow-up examinations than patients without or with low-grade OA (MEPS: 85 versus 65 points, *p* = 0.014; OES: 83 versus 63 points, *p* = 0.030, QuickDASH: 14 versus 43 points; *p* = 0.011). Moreover, patients with radiologic signs of persistent instability had significantly poorer functional outcome in MEPS (83 versus 63 points, *p* = 0.031) and OES (85 versus 59 points, *p* = 0.048).

### Impact of surgical treatment of the radial head and coronoid process

In cases involving a comminuted fracture of the proximal radius that were not suitable for partial resection, there were no significant differences between open reduction and internal fixation (ORIF) and radial head arthroplasty regarding MEPS (*p* = 0.813), OES (*p* = 0.815), QuickDASH (*p* = 0.512), postoperative pronation/supination arc (*p* = 0.212), post-traumatic OA (*p* = 0.398), or clinically relevant HO (*p* = 0.069).

**Fig. 5:**
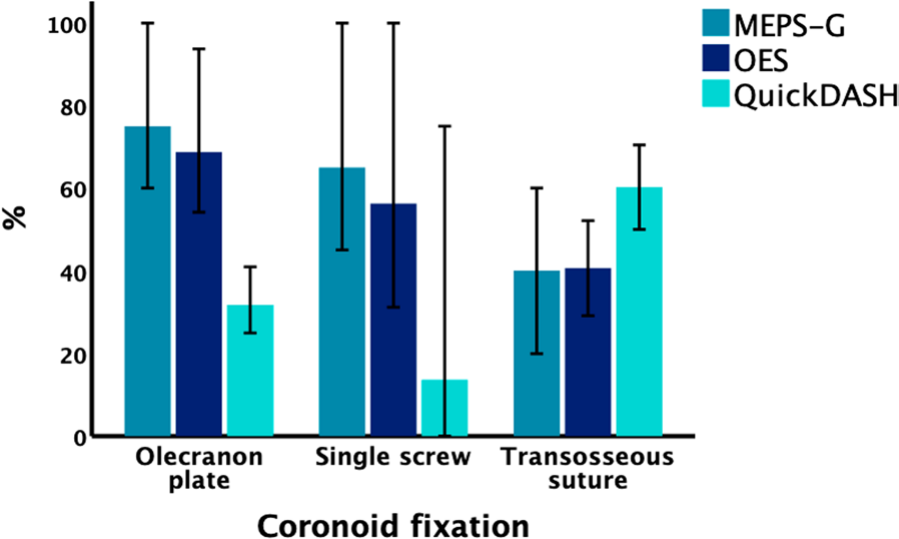
Functional outcome scores of TUBC cases depending on fixation strategy of the coronoid base fragment. There was a trend toward poorer outcome when sutures were used, but differences were not statistically significant. Error bars represent 95% CI. *CI* confidence interval, *OA* osteoarthritis, *MV* Monteggia-variant, *TO* Trans-olecranon, *TUBC* Trans-ulnar basal coronoid

In TUBC injuries, there was a tendency toward clinically relevant poorer outcomes (Fig. 5) when the coronoid fragment could not be adequately fixed with screws and was instead treated using transosseous sutures. In these cases, the MEPS showed a median of 60 versus 73 points (*p* = 0.383), the OES a median of 52 versus 69 points (*p* = 0.311), and the QuickDASH a median of 50 versus 26 points (*p* = 0.386).

Moreover, higher-grade osteoarthritis (OA) was observed in 6 of 19 elbows (32%) in which the coronoid was fixed with screws, either through the locking plate or with additional separate screws. In comparison, higher-grade OA was observed in three of five elbows in which the coronoid was fixed using transosseous sutures (*p* = 0.243).

### Revision surgeries

Of the 24 patients who were available for FU, 7 (29%; six TUBC, one MV) needed revision surgery. Of the six patients (40%) in the TUBC group, three were revised due to persistent instability, two received arthrolysis and HO resection, and one received revision for non-union. One additional patient with a TUBC injury suffered from neurapraxia of the radial nerve, which was treated non-operatively. Patients who required surgical revision had significantly greater impairment of arm function compared with those who did not undergo revision surgery (QuickDASH median 45 versus 21 points, *p* = 0.045).

## Discussion

This investigation presents midterm outcome data for trans-ulnar fracture–dislocations of the elbow classified according to the coronoid-centric Mayo classification system in a retrospective cohort. The key findings of this study are:

- Trans-ulnar basal coronoid (TUBC) fracture–dislocations represent the most frequent subtype among adult trans-ulnar elbow fracture–dislocations.
- Post-traumatic osteoarthritis (OA) occurs significantly more often after TUBC injuries, and higher grades of OA are associated with poorer functional outcomes.
- Functional outcomes following TUBC injuries are not inherently worse than those of Monteggia-variant (MV) or trans-olecranon (TO) fracture–dislocations; however, favorable results appear to depend critically on sufficient fixation of the basal coronoid fragment.

Fracture–dislocations of the elbow involving the coronoid base have long been recognized as the most severe form of trans-ulnar injury, regardless of the classification system applied.

O’Driscoll identified basal coronoid subtype II fracture–dislocations as particularly complex and unstable. Similarly, other authors have reported that injuries involving the coronoid base, commonly described as Jupiter type IIA or IID lesions, are associated with poorer outcomes and higher complication rates [23]. These findings led Barlow et al. [18] to propose a new Mayo classification system, defining trans-ulnar basal coronoid (TUBC) fracture–dislocations as a distinct injury entity, separate from Monteggia-variant (MV) and trans-olecranon (TO) fracture–dislocations.

From a classification standpoint, the Mayo system has demonstrated excellent inter- and intra-rater reliability [18] and appears to capture relevant prognostic differences by focusing on coronoid involvement. Earlier classification systems, including Bado’s Monteggia classification [8] with Jupiter’s subclassification of posterior Monteggia fracture–dislocations [9], the Montecranon system [14], the CURL classification [11], and the Wrightington classification [15], provided important biomechanical and conceptual insights. However, their complexity, limited applicability in certain cases, and unclear prognostic value has likely limited their widespread clinical use. In a systematic review of all published trans-ulnar fracture–dislocations classified according to the Mayo classification system [5], as well as in a retrospective single-center study [20], Nieboer et al. reported that TUBC injuries were associated with poorer functional scores and reduced arcs of motion compared with other subtypes. These findings align with the results of the present study, which also showed inferior outcomes in TUBC cases.

The reasons for these inferior results remain unclear. Possible explanations include the greater surgical complexity of TUBC injuries and a tendency to underestimate their severity during preoperative planning. In these cases, restoration of anterior stability is paramount. The coronoid process serves as the primary anterior buttress of the elbow, resisting posterior translation and varus stress [24]. Anterior stability depends mainly on the coronoid and the radial head, which have a comparable articulating surface areas although the radial head is exposed to higher loads [25]. Therefore, restoration of the radial column is likely decisive for treatment success in coronoid-deficient injuries. This may explain why radial head arthroplasty is often preferred over osteosynthesis in these fracture–dislocations. However, Klug et al. [26] demonstrated that the management of radial head fractures significantly influenced outcomes in trans-ulnar elbow fracture–dislocations. In their study, radial head arthroplasty was associated with inferior outcomes compared with reconstruction or resection. This observation could not be reproduced with statistical significance in our cohort. Differences in patient selection, injury severity, and surgical indications may account for this discrepancy.

Bagga et al. [11] identified coronoid reconstruction as the most important predictor of outcome in complex elbow fracture–dislocations, outweighing both olecranon and radial head fixation. It is well established that insufficient fixation or structural deficiency of the coronoid process, particularly in cases of severe comminution, results in persistent elbow instability [27, 28]. The findings of the present study strongly support this concept.

In our cohort, functional outcomes tended to be better when the basal coronoid fragment was large enough to allow stable fixation with lag screws, either through the olecranon plate or as independent screws. Conversely, outcomes were poorer when fixation relied on transosseous sutures. Post-traumatic OA was also more frequent in these cases, although this difference did not reach statistical significance. Notably, in this single-center cohort, no patient underwent an additional medial approach for direct fixation of the anteromedial coronoid facet, and no comminuted coronoid fracture was treated with buttress plating. Instead, coronoid reduction was achieved either indirectly through the basal ulnar fracture or via a deep lateral approach, the latter used in 27 of 37 cases with associated radial head fractures. However, when the coronoid fragment does not extend dorsally, a medial approach with lag screw or buttress plate fixation is recommended [29, 30]. In cases of severe comminution or coronoid deficiency, reconstructive strategies using structural allograft bone blocks or autologous osteochondral fragments from the radial head have also been described and may represent valuable alternatives [27, 28, 30, 31].

Several limitations of this study must be considered. First, the substantial loss to follow-up limits generalizability, although baseline characteristics did not differ significantly between patients with and without follow-up. Additionally, some patients died before follow-up assessment, which may reduce applicability of the results to older populations. The retrospective study design and reliance on existing medical records introduce potential selection bias. In addition, multiple surgeons were involved, and surgical decision-making was not standardized, particularly with respect to coronoid fixation techniques and radial head treatment. These factors may have influenced comparisons between injury types. Furthermore, radiographic follow-up was variable and predominantly short-term, with a median duration of 31 months, which may underestimate late degenerative changes. Finally, the relatively small cohort size, especially for Monteggia-variant injuries, limited statistical power. This likely contributed to the absence of statistically significant differences despite clinically relevant trends exceeding minimal clinically important differences. This limitation reflects the rarity of these injuries, with approximately five cases per year at our institution, consistent with previously reported incidences [14].

In conclusion, the present data support the hypothesis that TUBC lesions represent a high-risk subgroup among trans-ulnar fracture–dislocations of the elbow. Careful preoperative assessment and meticulous surgical planning are essential. While TO fracture–dislocations generally result in the most favorable functional and radiographic outcomes, good results can also be achieved in TUBC injuries when stable fixation of the coronoid base is accomplished. In contrast, insufficient restoration of the coronoid base appears to be associated with inferior outcomes and higher rates of post-traumatic degeneration. Larger, multi-center prospective studies are needed to identify predictors of treatment failure and to refine surgical strategies for these complex injuries.

## Data Availability

The datasets used and/or analyzed during the current study are available from the corresponding author on reasonable request for a period of 5 years.

## Abbreviations

EQ-5D-5L: European Quality of Life Five-Dimension, Five-Level Version
FU: Follow-up
HO: Heterotopic ossification
MEPS: Mayo Elbow Performance Score
MV: Monteggia-variant
OA: Osteoarthritis
OES: Oxford Elbow Score
QuickDASH: Quick Disabilities of Arm, Shoulder and Hand Questionnaire
TO: Trans-olecranon
TUBC: Trans-ulnar basal coronoid
